# Pharmacoeconomic effect of compliance with pharmacist’s intervention based on cancer chemotherapy regimens: a cohort study

**DOI:** 10.1186/s40780-014-0007-y

**Published:** 2015-03-05

**Authors:** Makoto Hayashi, Akimasa Yamatani, Hiromu Funaki, Kenichi Miyamoto

**Affiliations:** Department of Pharmacy, National Hospital Organization Kanazawa Medical Center, 1-1 Shimoishibiki-machi, Kanazawa, Ishikawa 920-8650 Japan; Department of Medicinal Informatics, Graduate School of Medical Science, Kanazawa University, 13-1 Takara-machi, Kanazawa, Ishikawa 920-8641 Japan

**Keywords:** Pharmacoeconomics, Pharmacist intervention, Regimen management

## Abstract

**Background:**

It is important for pharmacists to manage cancer chemotherapy regimens in order to achieve safe treatment. We examined whether there was a useful pharmacoeconomic benefit of compliance the exclusion criteria of neutropenia, and the importance of a pharmacist's intervention was considered.

**Methods:**

A prospective observational cohort study was conducted at a community-based medical center. Among 374 patients who received chemotherapy between April 2010 and March 2011, 108 patients developed neutropenia and pharmacists recommended suspension of chemotherapy. These patients were divided into a group in whom chemotherapy was suspended (complying group) and a group in whom it was continued (non-complying group). Then the relative dose intensity (RDI) was compared between the two groups, and medical expenses related to the treatment of neutropenia (neutropenia-related costs: NRC) were compared. Analysis was carried out from the perspective of the health insurance provider, so only the direct medical costs were evaluated.

**Results:**

There was a significant difference of the RDI between a complying group (85.2 ± 10.0%) and a non-complying group (79.3 ± 15.0%) (P = 0.021). The average NRC per patient showed a significant difference between the two groups (complying group: 1,944 ± 412 dollars, non-complying group: 4,394 ± 837 dollars, P = 0.044). The economic effect over one year was 54,205 dollars.

**Conclusion:**

The present findings suggest that ensuring compliance with chemotherapy regimens (including the criteria for neutropenia) is effective from a pharmacoeconomic perspective. Accordingly, pharmacists should intervene as required to improve regimen compliance.

## Background

In 1998, the concept of the regimen was proposed by the American Society of Health System Pharmacists (ASHP), American Medical Association (AMA), and the American Nurses Association (ANA), in order to prevent medication errors during anticancer chemotherapy [1]. Guidelines for describing all aspects of chemotherapy regimens, including the treatment protocols, order forms, and product labels, have been proposed by the ASHP and others. These standards for chemotherapy regimens are broadly applicable and can be adopted by a wide range of institutions. Clear and unambiguous medication orders and consistent descriptions of treatment are important. Treatment plans and orders should contain enough information to allow health care providers from diverse disciplines to compare them with published treatment regimens and investigational protocols, and must therefore include planned dosages and schedules expressed in patient-specific units. It is important for pharmacists to be involved in the management of anticancer regimens so that chemotherapy is performed safely, and there have been several reports that regimen management by pharmacists reduces medication errors [2-6].

Dose escalation and shortening of the interval between courses are two approaches to dose intensification that are based on mathematical modeling [7-10]. The Goldie-Coldman hypothesis predicts that delivering higher doses of anticancer agents will reduce the survival of resistant clones [11,12], while the Norton-Simon hypothesis goes beyond previous theories by incorporating the concept of chemotherapy schedule [12]. These hypotheses both highlight the role of dose and schedule, and have significantly influenced oncology practice. Relative dose intensity (RDI) is an index of the intensity of a particular chemotherapy regimen [13], and is thought to be an appropriate index of the curative effect of anticancer treatment. Patients receiving chemotherapy often experience delay of administration and dose reduction, leading to a decrease of the RDI. Dosage are often adjusted because of neutropenia/febrile neutropenia, since neutropenia is one of the major side effects of chemotherapy and is an exclusion criterion in many regimens. When a patient develops neutropenia, in addition to decreasing the RDI by prolonging the interval between courses, administration of granulocyte colony-stimulating factor (G-CSF) may be needed [14]. Under these circumstances, patients often not only receive inadequate treatment, but medical costs are increased. Thus, it is desirable to avoid neutropenia and to reduce medical costs by the pharmacist intervention in regimen management. However, no reports about the pharmacoeconomic benefits of pharmacist intervention in the management of anticancer chemotherapy have been published.

Accordingly, we attempted to show that compliance with pharmacist intervention based on the cancer chemotherapy regimen is important and has a pharmacoeconomic effect.

## Methods

### Regimen checking by pharmacists

**Figure 1:**
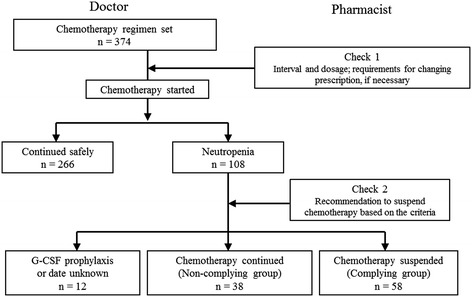
**Chemotherapy flow and regimen checking by pharmacists.** The pharmacist performed the regimen check and patients were classified into the complying group and the non-complying group depending on whether the doctor followed the pharmacist’s advice.

The procedure for regimen checking by pharmacists is shown in Figure 1. First, the attending doctor selected chemotherapy for a patient. Before chemotherapy was started, a pharmacist reviewed the regimen (administration schedule and drug dosages) and asked the doctor to alter it if any problems were detected (Check 1). Before the start of chemotherapy, if a request for testing the neutrophil count was not provided by the doctor, a pharmacist ordered the test instead. If the neutrophil count met the exclusion criteria, the pharmacist issued a warning. If the doctor decided to perform chemotherapy anyway, the pharmacist issued another warning (Check 2).

The exclusion criteria for each regimen were set by our hospital’s regimen screening committee, which included a chemotherapy specialist. The criteria for the neutrophil count were based on the product information for each medication and on data from clinical trials [15,16].

### Study design

A prospective observational cohort study was conducted at a community-based medical center. We enrolled 374 patients who received chemotherapy between April 2010 and March 2011 at the National Hospital Organization Kanazawa Medical Center in Japan. After starting chemotherapy, 108 patients developed neutropenia, and pharmacists recommended suspension of chemotherapy based on the regimen criteria (Table 1).

**Table 1:** **Neutrophil count for suspending treatment with each anticancer agent**

**Anticancer agent**	**Neutrophil count (/μl)**
5-fluorouracil	<1,000
Bendamustine	<1,000
Carboplatin	<1,000
Cisplatin	<1,000
Cyclophosphamide	<1,000
Cytarabine	<1,000
Docetaxel	<1,000
Doxorubicin	<1,000
Etoposide	<1,000
Gemucitabine	<1,000
Ifosfamide	<1,000
Irinotecan	<1,500
Methotorexate	<1,000
Oxaliplatin	<1,500
Paclitaxel (tri weekly)	<2,000
Paclitaxel (weekly)	<1,500
Pirarubicin	<1,000
Pemetrexed	<1,500
Vinorelbine	<2,000

These 108 patients were divided into a complying group (n = 58) for whom chemotherapy was suspended, a non-complying group (n = 38) who continued chemotherapy, and another group (n = 12) who received G-CSF prophylaxis, stopped chemotherapy for reasons other than neutropenia, or failed to attend hospital regularly.

We performed a retrospective investigation of factors related to cost effectiveness by performing comparisons between the complying and non-complying groups.

### Analysis of cost effectiveness

Costs were investigated by calculating the neutropenia-related direct medical costs (NRC). These costs were computed from hospital statements and included drug costs (G-CSF, etc.) plus all expenses for medical examination and management.

The relative dose intensity (RDI) was employed as an index of effective delivery of chemotherapy. RDI was calculated as the ratio of actual total dose intensity (ATDI) to the planned total dose intensity (PTDI), and was expressed as a percentage:

$$ \mathrm{R}\mathrm{D}\mathrm{I}\left(\%\right)=\mathrm{ATDI}/\mathrm{PTDI}\times 100. $$

PTDI was defined as the average planned dose intensity over the entire treatment period across all of the chemotherapy agents administered. ATDI was calculated by setting the scheduled regimen dose as 100%.

NRC and RDI were compared between the complying and non-complying groups. Analysis was carried out from the perspective of the health insurance provider, so only medical costs were evaluated.

### Statistical analysis

Results are expressed as the mean ± SD or as numbers (%). For comparison of patient characteristics, age was compared by the unpaired *t*-test, while sex, prior chemotherapy, Eastern Cooperative Oncology Group performance status, adjuvant chemotherapy, and clinical department were evaluated by Fisher’s exact test.

The neutrophil count, RDI, duration of G-CSF therapy, and NRC were compared by the unpaired *t*-test, while the rate of G-CSF use was evaluated by Fisher’s exact test.

Multiple regression analysis was performed to determine the relationship between NRC and each of the above variables. Correlations between NRC and each variable were investigated by univariate analysis, and variables showing P < 0.05 were included in multiple regression analysis. To determine the factors influencing the NRC, stepwise selection was performed with the following independent variables: regimen compliance, neutrophil count, and leukemia. A two-sided statistical test was used in all analyses and P <0.05 was considered to indicate significance. Statistical software (Dr SPSS for Windows, Version 5.0) was employed for these analyses.

This study was approved by the research ethics committee of Kanazawa Medical Center (No. 2012–002) and was performed according to the Declaration of Helsinki.

## Results

### Patients

A total of 108 patients developed neutropenia corresponding to the criteria for suspension of treatment. Among them, 12 patients who received G-CSF prophylaxis were excluded. The remaining 96 patients were divided into a complying group (n = 58), which suspended chemotherapy according to the pharmacist's recommendation, and a non-complying group (n = 38) that continued chemotherapy against the pharmacist's recommendation. Then costs related to treating neutropenia were compared between the complying group and the non-complying group. Patient characteristics are listed in Table 2. There were no differences of clinical factors between the two groups. A classification of the subjects by chemotherapy regimen is shown in Table 3. There were no significant differences between the two groups.

**Table 2:** **Characteristics of the complying and non-complying groups**

	**Total n = 96**	**Complying group n = 58**	**Non-complying group n = 38**	**P value**
Age (years)	68.6 ± 10.8	68.2 ± 10.2	69.3 ± 11.7	0.627^1)^
Male/female	44/52	27/31	17/21	0.999^2)^
History of chemotherapy (yes/no)	25/71	17/41	8/30	0.477^2)^
ECOG PS (0-1/2)	93/3	57/1	36/2	0.560^2)^
Adjuvant chemotherapy (yes/no)	7/92	6/52	1/37	0.238^2)^
Clinical department (number, %)				
Surgery	25 (26.0)	18 (31.0)	7 (18.4)	
Hematology	31 (32.3)	6 (10.3)	25 (65.8)	
Respiratory medicine	19 (19.8)	19 (32.8)	0 (0.0)	
Gastroenterology	11 (11.5)	7 (12.1)	4 (10.5)	0.098^3)^
Gynecology	4 (4.2)	4 (6.9)	0 (0.0)	
Urology	5 (5.2)	4 (6.9)	1 (2.6)	
Dental surgery	1 (1.0)	0 (0.0)	1 (2.6)	
Neutrophil count (/mL)	730±245	746±286	708±240	0.37^1)^

Data are expressed as the mean SD or number (%).^1)^unpaired t-test, ^2)^Fisher’s exact test, ^3)^Mann–Whitney test. ECOG PS, Eastern Cooperative Oncology Group performance status. G-CSF, Granulocyte colony-stimulating factor.

**Table 3:** **The classification by chemotherapy regimens**

**Chemotherapy regimen**	**Total n = 96**	**Complying group n = 58**	**Non-complying group n = 38**
5-flourouracil	1	1	0
ABVD	2	0	2
AC	7	6	1
Bendamustine	3	0	3
B-mab+CBDCA+PAC	1	1	0
B-mab+mFOLFOX6	3	2	1
CBDCA+GEM	2	2	0
CBDCA+VP16	2	2	0
CHOP	5	1	4
DeVIC	2	1	1
DOC	2	0	2
DOXIL	2	2	0
GC	1	1	0
GEM	12	12	2
High dose-AraC	2	0	2
Hign dose MTX	2	0	2
Irinotecan	2	2	0
Pemetrexed + CBDCA	3	3	0
Pemetrexed	1	1	0
PAC+GEM	2	2	0
R-CHOP	3	0	3
Rituximab	1	1	0
R-THPCOP	10	4	6
S1+DOC	2	2	0
S1+CDDP	1	1	0
S1+GEM	8	6	2
SOX	4	1	3
THPCOP	2	0	2
VNR + HER	1	0	1
CBDCA+PAC	1	1	0
Weekly PAC	1	1	0
Weekly PAC+ HER	2	1	1

ABVD: doxorubicin+ bleomycin+ vincristine+ dacarbazine, B-mab: bevacizumab, CBDCA: carboplatin, PAC: paclitaxel mFOLFOX 6: 5-fluorouracil+ levofolinate+ oxaliplatin, DOC: docetaxel, GEM: gemcitabine, VP16: etoposide, CHOP: cyclophosphamide+ doxorubicin+ vincristine + predonizorne, DeVIC: carboplatin+etoposide+ifosfamide, GC: emcitabine+ cisplatin, AraC: cytarabine, MTX: methotrexate, R: rituximab, THPCOP: rituximap+cyclophosphamide+pirarubicin+vincristine + Predonizorne, S1: Tegafur+ gimeracil+ oteracil, CDDP: cisplatin, SOX: S1+ oxaliplaton, VNR: vinorelbine, HER: trastuzumab.

### Analysis of Cost effectiveness (Table 4)

There was a significant difference of RDI between the complying group (85.2 ± 10.0%) and the non-complying group (79.3 ± 15.0%) (P = 0.021), with the RDI being significantly higher in the complying group. Use of G-CSF for neutropenia was similar, since it was employed in 56.3% of the complying group and 60.5% of the non-complying group (P = 0.08). However, the duration of G-CSF treatment was significantly shorter in the complying group (10.7 ± 14.8 days) than in the non-complying group (20.7 ± 22.3 days, P = 0.049).

**Table 4:** Analysis of cost effectiveness

	**Total n = 96**	**Complying group n = 58**	**Non-complying group n = 38**	***P*** **value**
Number using G-CSF	56 (58.3%)	33 (56.9%)	23 (60.5%)	0.857^2)^
Duration of G-CSF (days)	14.8 ± 10.8	10.7 ± 14.8	20.7 ± 22.3	0.049^1)^
Total cost G-CSF (days)	279,731	112,763	166,968	
Cost of outpatient visit (dollar)	26,131	9,306	16,825	
Cost of hospitalization (dollar)	253,600	103,457	150,143	
NRC (dollar/patient)	2,914 ± 594	1,944 ± 412	4,394 ± 837	0.044^1)^
RDI (%)	82.9 ± 13.5	85.2 ± 10	79.3 ± 15	0.021^1)^

Data are expressed as the mean (SD) or number (%).^1)^unpaired t-test, ^2)^Fisher’s exact test. ECOG PS, Eastern Cooperative Oncology Group performance status. NRC, neutropenia-related costs. RDI, relative dose intensity.

The total neutropenia-related cost (NRC) of treatment, including the costs for outpatient visits and for hospitalization, was 279,731 dollars. It was 112,763 dollars in the complying group and 166,968 dollars in the non-complying group. The average NRC per patient showed a significant difference between the two groups (complying group: 1,944 ± 412 dollars, non-complying group: 4,394 ± 837 dollars, P = 0.044). The economic effect over one year was 54,205 dollars.

### Factors influencing the NRC

The results of univariate analysis of nine variables are displayed in Table 5. Variables that showed a significant influence in this analysis were regimen compliance, the neutrophil count, and leukemia (P > 0.05). Multivariate analysis was performed with these three variables identified by univariate analysis and the results are shown in Table 6. Only regimen compliance and leukemia had a significant independent influence on the NRC (adjusted r2 = 0.69, P <0.001).

**Table 5:** **Correlation coefficients between NRC and other parameters (univariable analysis)**

**Variable**	**Correlation coefficient (r)**	**P value**
Regimen compliance (yes:1)	-0.298	0.026^1)^
Age (years)	0.115	0.218^2)^
Male (yes:1)	-0.003	0.980^1)^
Adjuvant therapy (yes:1)	-0.256	0.057^1)^
History of chemotherapy (yes:1)	0.155	0.254^1)^
ECOG PS (0-1/2)	-0.012	0.929^1)^
Neutrophil count (/μL)	-0.197	0.041^2)^
Malignant lymphoma (yes:1)	0.239	0.076^1)^
Leukemia (yes:1)	0.457	<0.001^1)^

Spearman’s correlation coefficient ^1)^, Pearson’s correlation coefficient ^2)^ECOG PS, Eastern Cooperative Oncology Group performance status.

**Table 6:** **Predictors of the NRC by multiple regression analysis**

**Variable**	**β**	**T-value**	**P value**	**VIF**
Regimen compliance (yes:1)	-0.237	-2.915	0.005	1.025
Neutrophil count (/μL)	-0.059	-0.685	0.384	1.152
Leukemia (yes:1)	0.761	8.917	<0.001	1.129

The adjusted coefficient of determination (*R* ^*2*^) was 0.66.

β: standardized partial regression coefficient, VIF: variance inflation factor.

## Discussion

Regimen compliance, including avoidance of neutropenia, is important for safe performance of chemotherapy. In the present study, we examined the pharmacoeconomic effect of compliance with pharmacist intervention based on cancer chemotherapy regimens. We found that the NRC was significantly lower in the complying group who discontinued chemotherapy according to the regimen compliance. The reason for the higher NRC in the non-complying group seemed to be administration of G-CSF for a longer period than in the complying group. The expenses that we investigated in this study not only included the cost of G-CSF itself, but also the other medical costs for treatment of neutropenia.

In Japan, when a fixed level of medical expenses for high-cost medical care is exceeded, payment by the patient is reduced. The results of this study suggest that regimen management by pharmacists not only contribute to reducing payments by the patient but also to reduction of overall medical costs [2,3].

There have already been some reports that pharmacist intervention can contribute to reducing medical costs. Moore et al. [17] reported that intervention by pharmacists targeting the medication of high-risk patients could reduce total costs and treatment costs for diseases such as diabetes, hypertension, dyslipidemia, depression, and asthma. In addition, Yu et al. [18] reported that educational intervention by pharmacists showed excellent cost effectiveness for Type 2 diabetes mellitus. The present study provided evidence that pharmacist intervention in anticancer chemotherapy can also improve cost effectiveness.

On the other hand, pharmacist intervention should not adversely influence the curative effect of chemotherapy. It has been shown that the RDI is an important indicator of curative effect [4-6]. In this study, the RDI of the complying group was clearly higher than that of the non-complying group. An RDI > 85% appears to improve the long-term outcome of chemotherapy [19], and the RDI of the complying group was 85.2%. Because a high RDI seems to prolong overall survival and the time to progression, the usefulness of regimen compliance is suggested.

Chemotherapy was continued despite the warning from the pharmacist in 38 patients for the following reasons. The attending doctor hoped for a curative effect in 21 patients. In another 9 patients, the neutrophil count was only slightly below the borderline for suspending treatment. In the remaining 8 patients, the reason could not be determined. Thus, continuation of treatment against the advice of the pharmacist was based on the judgment of the attending doctor, and selection bias regarding continuation of chemotherapy was not identified.

Multivariate analysis revealed that factors influencing the NRC were regimen observance and treatment for leukemia. Patients with hematologic malignancies, particularly leukemia and lymphoma, often show delayed recovery from severe neutropenia due to their underlying disease and the need for strong chemotherapy, and leukemia patients may achieve CR even if treatment is started when the neutrophil count is decreased. In this study, there were much more patients with hematologic malignancies in the non-complying group than in the complying group, which could be a reason why G-CSF was administered for longer in the non-complying group. However, even after adjusting for leukemia and lymphoma, regimen observance still had an independent influence on the NRC.

There were some limitations of this study. The first limitation was that the attending doctor revised the anticancer drug dosages after next courses. In addition, the timing of resuming chemotherapy may have differed between inpatients and outpatients. Although inpatients can undergo daily blood tests, outpatients usually undergo testing on a weekly basis. However, the number of outpatients was similar in the complying group (n = 12, 20.7%) and the non-complying group (n = 10, 26.3%), possibly reflecting the routine use of G-CSF.

The second limitation is that the G-CSF treatment period depended on the neutrophil count, which was not necessarily checked every day, so the possibility of a change in NRC based on the frequency of testing cannot be denied.

The third limitation of this study is that the RDI was used as the index of the effect of chemotherapy. A better index would have been overall survival, which we hope to employ in a future investigation.

Further, the index of cost was set as NRC in the present study. Although suspension of chemotherapy is not only due to neutropenia, this study could not assess whether other adverse events influenced the suspension of the chemotherapy.

Despite the above limitations, we conclude that regimen compliance include neutropenia for chemotherapy has a beneficial pharmacoeconomic effect and pharmacists have played the role important for a regimen management. Although it has already been reported that regimen management by pharmacists contributes to safety, this is the first evidence that pharmacist's recommendation to suspend chemotherapy based on the criteria for neutrophil counts also has an economic benefit.

## Conclusions

The present findings suggest that ensuring compliance with chemotherapy regimens (including the criteria for neutropenia) is effective from a pharmacoeconomic perspective. Accordingly, pharmacists should intervene as required to improve regimen compliance.
