# Erratum to: Pharmacoeconomic effect of compliance with pharmacist’s intervention based on cancer chemotherapy regimens: a cohort study

**DOI:** 10.1186/s40780-016-0037-8

**Published:** 2016-01-26

**Authors:** Makoto Hayashi, Akimasa Yamatani, Hiromu Funaki, Kenichi Miyamoto

**Affiliations:** Department of Pharmacy, National Hospital Organization Kanazawa Medical Center, 1-1 Shimoishibiki-machi, Kanazawa, Ishikawa 920-8650 Japan; Department of Medicinal Informatics, Graduate School of Medical Science, Kanazawa University, 13-1 Takara-machi, Kanazawa, Ishikawa 920-8641 Japan

Unfortunately, the original version of this article [1] contained two errors. The symbol “Equal contributors” was incorrectly attributed to all authors and the email address from the corresponding author was also included incorrectly. The symbol “Equal contributors” has been removed and the correct email address of the corresponding author can be found in this erratum.
